# Prediction and Analysis of Quorum Sensing Peptides Based on Sequence Features

**DOI:** 10.1371/journal.pone.0120066

**Published:** 2015-03-17

**Authors:** Akanksha Rajput, Amit Kumar Gupta, Manoj Kumar

**Affiliations:** Bioinformatics Centre, Institute of Microbial Technology, Council of Scientific and Industrial Research (CSIR), Sector 39-A, Chandigarh-160036, India; University of Alberta, CANADA

## Abstract

Quorum sensing peptides (QSPs) are the signaling molecules used by the Gram-positive bacteria in orchestrating cell-to-cell communication. In spite of their enormous importance in signaling process, their detailed bioinformatics analysis is lacking. In this study, QSPs and non-QSPs were examined according to their amino acid composition, residues position, motifs and physicochemical properties. Compositional analysis concludes that QSPs are enriched with aromatic residues like Trp, Tyr and Phe. At the N-terminal, Ser was a dominant residue at maximum positions, namely, first, second, third and fifth while Phe was a preferred residue at first, third and fifth positions from the C-terminal. A few motifs from QSPs were also extracted. Physicochemical properties like aromaticity, molecular weight and secondary structure were found to be distinguishing features of QSPs. Exploiting above properties, we have developed a Support Vector Machine (SVM) based predictive model. During 10-fold cross-validation, SVM achieves maximum accuracy of **93.00%**, Mathew’s correlation coefficient (MCC) of **0.86** and Receiver operating characteristic (ROC) of **0.98** on the training/testing dataset (T^200p+200n^). Developed models performed equally well on the validation dataset (V^20p+20n^). The server also integrates several useful analysis tools like “*QSMotifScan”*, *“ProtFrag”*, *“MutGen” *and *“PhysicoProp”*. Our analysis reveals important characteristics of QSPs and on the basis of these unique features, we have developed a prediction algorithm “QSPpred” (freely available at: http://crdd.osdd.net/servers/qsppred).

## Introduction

Bacteria communicate and coordinate their behavior by the use of signal molecules secreted by self, other bacteria or both [1]. Quorum sensing (QS) is a biological phenomenon through which bacteria communicate with each other by sending and receiving these chemical signals [1,2]. They use this phenomenon to assess the size of their population by measuring the concentration of these signals [3,4]. This phenomenon was first described in a Gram-positive bacteria, *Streptococcus pneumonia*, in which competence was supposed to be controlled by a hormone-like extracellular peptide [5]. It was later discovered in two luminous Gram-negative marine bacterial species, *Vibrio fischeri* and *Vibrio harveyi* [6]. QS helps bacteria in their survival through biofilm formation, virulence, swarming motility, genetic competence, bioluminescence and sporulation [1,3,7].

Quorum sensing phenomenon is driven by the involvement of signaling molecules that are oligopeptides (or autoinducing peptides (AIPs) [1] or Quorum sensing peptides (QSPs)) in Gram-positive bacteria and acylated homoserine lactone (AHL) in Gram-negative bacteria [1]. In Gram-positive bacteria, QSPs are secreted into the extracellular space by ATP-binding cassettes (ABC transporters) and accumulates in high density, after reaching a threshold it initiates a signaling cascade of events via two-component system [8] or by direct binding to transcription factorafter peptide import [9]. After the detection of QSPs by bacteria, response regulator or transcriptional factor get activated and stimulates change in target gene expression [9]. QSPs are species specific having varied lengths that may adopt a linear or cyclic conformation after post-translational modifications [4]. Besides, QSP and AHL, other signaling molecules like Diketopiperazines (DKPs) and *Pseudomonas* quinolone signal (PQS) have also been reported in some bacteria [10,11].

Several QSPs have been reported to perform various functions in different clinically relevant bacteria. For example, biofilm formation regulated by AIP2 in *Staphylococcus epidermidis* [12]; mating response [13] and expression of pathogenicity-related extracellular protease in *Enterococcus faecalis* [14]. Further, natural competence is synchronized by peptide pheromone i.e., competence-stimulating peptides (CSPs) in *S*. *pneumonia* and *Streptococcus gordonii* [15]. Antimicrobial peptides (AMPs), namely, lantibiotics and bacteriocins are known to produced with the help of AIPs [16]. Besides Gram-positive bacteria, QSPs have lately been reported in Gram-negative bacteria *Escherichia coli* as linear pentapeptides responsible for programmed cell death [17]. The mechanism, importance and application of these peptides have been reviewed in several studies [1,18].

Targeting of QSPs may provide an alternative strategy to combat bacterial pathogenicity [19]. Quorum quenching (QQ) is an approach for disrupting the quorum-sensing mechanism. It can be achieved using small molecules, monoclonal antibodies and antagonists against the receptors [18,20]. For example, ambuic acid, a secondary fungal metabolite acts as an active inhibitor of cyclic peptides (also known as Quoromones) in Gram-positive bacteria [21]. Siamycin I, a secondary metabolite of actinomycetes inhibits quorum sensing in *E*. *faecalis* [22]. RNA III Inhibiting Peptide (RIP), a heptapeptide impedes *S*. *aureus* pathogenesis by disrupting quorum sensing mechanism [22]. AP4–24 H11, an anti-autoinducer monoclonal antibody, helps in hindering auto inducing peptide (AIP)-4 produced by *S*. *aureus* RN4850 [23].

Despite the immense importance of QSPs, only one database of experimental QSPs, Quorumpeps is available [7]. However, their detailed bioinformatics analysis is lacking. Therefore, in this study we have analyzed QSPs exploiting various peptide features, namely, amino acid compositions, amino acids positional preferences, motifs and important physicochemical properties. Additionally, distinctiveness of these peptides was used to develop an SVM based algorithm QSPPred for predicting unknown peptides as QSP or not.

## Materials and Methods

### Data collection

For this study, QSPs were extracted from Quorumpeps database [7] having 231 entries reported from 1955–2012. For subsequent period, we have searched PubMed and collected 10 more entries. From the total of 241 entries, 100% identical peptides (redundant) were removed and 220 unique experimentally verified QSPs were utilized for further analysis. The length of these QSPs varies from 5 to 48 with an average length of 12 amino acids.

For negative data set i.e. non-QSPs, we searched the literature and found only 5 experimentally validated entries. Due to lack of experimentally proven non-QSPs, two strategies were used for selecting negative datasets. Firstly, a negative dataset was extracted from UniProt. Query “Gram-positive bacteria NOT quorum sensing AND sequence length range 5 to 65” was used to obtain negative data equivalent to positive data set. We could extract only 215 peptides that were not involved in quorum sensing. In the case of inadequate experimentally proven negative dataset, this strategy of selecting a negative data set from UniProt has also been reported in previous studies [24,25]. From a total data of 440 (220p+220n), we have extracted 20 peptides each from positive and negative data sets by using random number generator to finally have a training/testing data set of 400 peptide sequences (T^200p+200n^) and a validation data set of 40 peptide sequences (V^20p+20n^). Secondly, each QSP sequence was scrambled using software (http://users.umassmed.edu/ian.york/Scramble.shtml) for generating equal number of negative dataset. Complete data 440 (220p+220sn) divided in training/test data set 400 peptide sequences (T^200p+200sn^) and validation data set of 40 peptide sequences (V^20p+20sn^) using random number generator.

### QSP properties

#### Amino acid composition

Amino acid composition (AAC) is an important feature to be explored in case of peptides and proteins. It represents the frequency of each amino acid in a sequence. Fraction of each amino acid can be calculated by the formula:

$$Fraction\;ofaminoacid(x)=\frac{Totalnumberofx}{sequencelength}$$

#### Amino acid residue position

Sequence logos are used which gives position specific frequency of amino acids in a sequence [24]. A stack of symbols represents each position in a sequence whereas stack height corresponds to the sequence conservation at that position. While relative frequency of each amino acid determines the height of the symbol within a stack. In this study we are using an extended form of WebLogo (http://www.twosamplelogo.org/), Two sample logo, that displays the difference between two sets of groups on the basis of a position-specific symbol composition [26].

#### Motif identification

Presence of specific motifs has been reported in different peptides [24,27]. Therefore, we have also explored motifs present in QSPs using MEME/MAST 4.9.1 [28]. Positive and negative QSPs were scanned to identify motifs at different E-values (10^-7^ to 10) and Positive Predictive Value (PPV) along with percent coverage was calculated as:

$$PPV=\frac{TP}{TP+FP}$$

$$\%\;coverage=\frac{TP}{TP+FN}\times 100$$

Where, TP, FP and FN are true positive, false positive and false negative respectively. These extracted motifs will be searched in other organisms using Bioinformatics Toolkit [29].

#### Physicochemical properties

The QSPs are functional peptides like others *viz*. antiviral peptides (AVPs) [30] and antimicrobial peptides (AMPs) [25] etc. Therefore, we have selected some of the properties reported earlier to analyze other functional peptides. These general properties like length, aromaticity, instability index, isoelectric point, molecular weight, α-helix, β-sheet, coil and hydrophobicity were used to see whether these differ between QSPs and non-QSPs. In addition, top physicochemical indices among 544 physicochemical properties from AAindex [31] that performed well during SVM based classification were also used for analysis of QSPs and non-QSPs.

Some basic physicochemical properties like isoelectric point and molecular weight have been estimated using ProtParam tool from ExPASy.

*Aromaticity*: ProtParam tool is utilized to calculate aromaticity. It is the relative frequency of aromatic amino acids present in any peptide/ protein [32]. It is calculated as:

$$Aromaticity=\sum_{i=1}^{20}\delta_{i}f_{i}$$

Where, f_i_ is a relative frequency of amino acids of kind i in peptide/protein and δ_i_ = 1 when the amino-acid is aromatic (Phe, Tyr, Trp) and δ_i_ = 0 otherwise.

*Instability index*: It is calculated by ProtParam tool. It is the measurement of the stability of peptide/protein. It is estimated as:

$$II=\;\frac{10}{L}\sum_{i=1}^{i=L-1}DIWV(x_{i}y_{i+1})$$

Where, L is the length of a sequence and 10 is a scaling factor. DIWV is the dipeptide instability weight value of a dipeptide starting at position I [33]. A sequence having II smaller than 40 is considered to be stable.

*Grand average of hydropathy (GRAVY)*: GRAVY Calculator (http://www.gravy-calculator.de/) is used to calculate the average hydropathy of sequence. It is the sum of hydropathy values [34] of all amino acids divided by the protein length.

*Secondary structure*: PSSpred V2 (Protein Secondary Structure PREDiction), a bioinformatics tool in I-TASSER [35] was used to predict secondary structure of sequences in our datasets. PSSpred V2 predicts the propensity of each amino acid to be in α-helix, β-sheet or coil conformation. We have evaluated these propensities in every sequence.

### Algorithm development

For developing prediction algorithm of QSPs, we utilized various sequence features of a peptide-like amino acid composition (AAC), dipeptide composition (DPC), binary patterns (N5C5Bin), physicochemical properties (Physico) and their hybrids. These properties have already been reported in various prediction methods [24,27].

### Support vector machine

A support vector machine is a supervised machine learning technique (MLT) used for both classification and regression analysis. SVM develops the predictive model by recognizing patterns in the training/testing data set which is used to assign categories to the unknown sequence [27]. For SVM implementation, variable peptide lengths were converted to fixed length patterns using several sequence properties. We have developed predictive models by applying SVM^*light*^version 6.02. Performance was optimized using RBF kernel on diverse g and c values.

### Ten-fold cross validation

We used 10-fold cross-validation to evaluate the performance of predictive models. In 10-fold cross-validation, complete data set is randomly divided into 10 sets, out of which 1 set (test) is tested by a model developed on the remaining 9 sets (training) and this process is iterated 10 times.

### Performance measures

Performance of various models in this study was computed using two modules, threshold dependent and threshold independent. In threshold dependent, we used the specificity, sensitivity, accuracy and Mathew's correlation coefficient (MCC) as calculated by the following equations:

$$Sensitivity=\frac{TP}{TP+FN}\times 100$$

$$Specificity=\;\frac{TN}{TN+FP}\times 100$$

$$Accuracy=\frac{TP+TN}{TP+FP+TN+FN}\times 100$$

$$MCC=\frac{\left(TP\times TN)-(FP\times FN\right)}{\sqrt{\left(TP+FP)(TP+FN)(TN+FP)(TN+FN\right)}}\times 100$$

Where, TP, TN, FP and FN are true positive, true negative, false positive and false negative respectively while MCC is Mathew's correlation coefficient.

In threshold independent parameter, we used ROC (Receiver Operating Characteristic) to assess performance of different SVM models. We created ROC plots showing area under curve (AUC) using ROCR [36] statistical package available in R.

## Results

### Evaluation of QSPs using different sequence properties

#### Analysis of QSPs using amino acids composition

**Fig 1:**
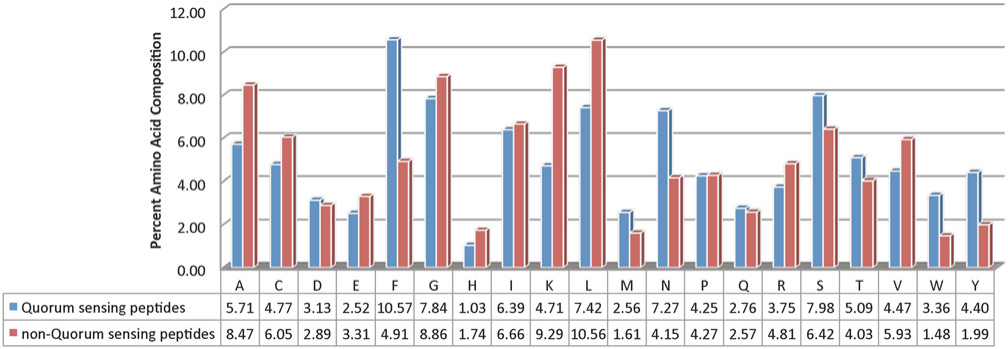
Amino acid compositional comparison of quorum sensing peptides (QSPs) and non-quorum sensing peptides (non-QSPs).

**Fig 2:**
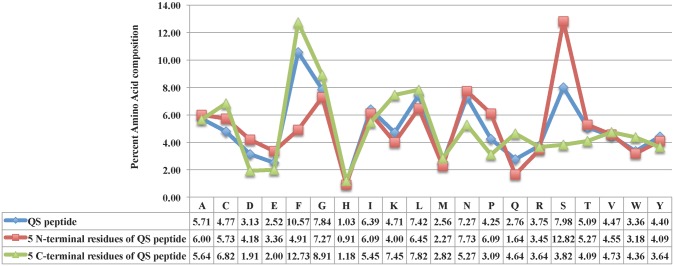
Amino acid compositional analysis of Quorum sensing peptides (QSPs). Comparison of percent amino acid composition of QSPs with their 5 N-terminal and 5 C-terminal residues.

To recognize whether certain types of residues are favored in QSPs, we compared amino acid residues present in QSPs and non-QSPs as shown in Fig. 1. In QSPs, aromatic amino acids were found to prefer as compared to non-QSPs like Trp, Tyr and Phe. In addition, the overall composition of QSPs was also compared with that of Gram-positive, Gram-negative bacteria proteome and proteins present in Swiss-Prot as shown in Fig. A in S1 File. Preference of certain amino acids in 5 N- and 5 C- terminal residues of QSPs were also checked. We observed that N-terminal contains small residues like Ser, Asn and Pro while less proportion of Gln, Phe and Lys. C-terminal has more propensities of large residues including Phe, Lys, Cys, Gln, Trp while less frequency of Asn, Ser, Pro, Asp as shown in Fig. 2.

### Analysis of QSPs using amino acids position

**Fig 3:**
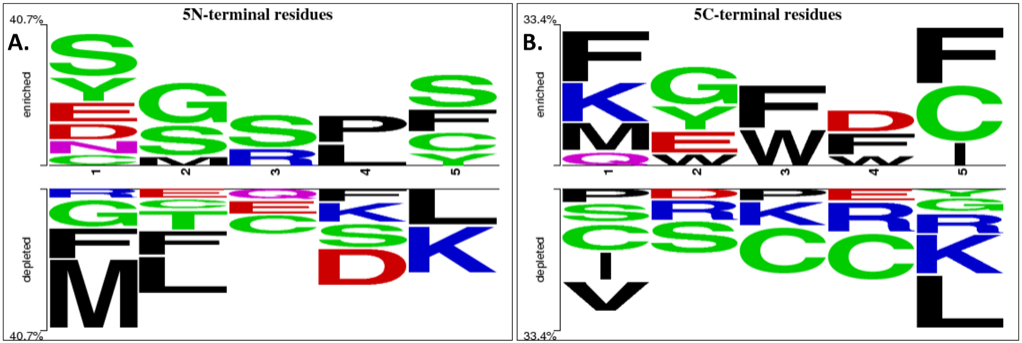
Two sample sequence logo. Figure depicting amino acid residues of Quorum sensing peptides (QSPs) at A) 5 N- terminal, B) 5 C- terminal.

To analyze which residues are favored at specific positions in QSPs, we examined the frequency of residues at both N and C-terminal using two sample sequence logos. Sequence logos of 5 amino acids of N and C terminus were generated as shown in Figs. 3A and 3B, respectively. Positional analysis of 5 N-terminal residues showed that Ser is found at maximum positions *viz* first, second, third and fifth while Gly is preferred at first and second positions in QSPs. Besides, some more residues like Asn and Pro were also found. Whereas at the C-terminal, Phe was preferred at first, third and fifth positions and Cys at fifth position.

### Identification of QSPs motif

We have extracted motifs from QSPs using the MEME software at E-values ranging from 10 to 10^-7^. At E-value 10, the positive predictive value (PPV) was 0.92 with percent coverage of 71.92%. At E-value 1, PPV increased to maximum 1.00 with a slight decrease in percent coverage to 65.07%. From E-value 0.1–10^-7^, the PPV remains 1.00 and percent coverage decreases to 20.55%_._ Detailed information of about QSPs motifs is provided in Table 1. List of the motifs at E- value 1 along with their regular expression are shown in Table 2.

**Table 1:** Motifs present at different E-values along with their corresponding positive predictive value (PPV) and percent coverage.

E-value	PPV	% Coverage
10	0.92	71.92
1	1	65.07
0.1	1	60.27
1.00E-02	1	54.11
1.00E-03	1	46.57
1.00E-04	1	29.45
1.00E-05	1	25.34
1.00E-06	1	22.60
1.00E-07	1	20.55

**Table 2:** Motifs list at E-value 1 along with their regular expression and number of sequences having respective motifs.

Motifs	Regular Expression	Number of motifs
Motif 1	LSTFFRLFNRSFTQA	20
Motif 2	[YP][NS][PTI][CF]GQ[YW][MF]	38
Motif 3	[TA]S[NS][IL][SV][KE]CVFS[FL]FKKC	8
Motif 4	E[SM]R[LI][SP][KR]I[LI][LR]DF	7
Motif 5	[DE]I[IL]IIVGG	7
Motif 6	LPYF[AF][GK][CH]L	5
Motif 7	[DS]SAC[VY][VW][GC]	7
Motif 8	G[LW]WE[DE][LI]L[YH]	3
Motif 9	DPITRQW	2

We searched nine QS motifs (obtained at E-value 1) using the pattern search tool of Bioinformatics Toolkit [29]. Maximum hits were obtained from Gram-positive bacteria namely, *Streptococcus mutans*, *E*. *faecalis*, *Carnobacterium maltaromaticum*, *Enterococcus faecium*, *S*.*pneumonia*, *Streptococcus oralis*, *B*. *subtilis*, *Streptococcus pseudopneumoniae*, etc. Hits were found in some Gram–negative bacteria like *Desulfatibacillum sps*, *Niastella koreensis*, *Campylobacter cuniculorum*, *Burkholderia sps*, *Vibrio nigripulchritudo*, etc. In addition, QS motif hits were also located in archea like *Methanobrevibacter sps* and fungus *Blumeria graminis*, *Arthroderma otae*, *Agaricus bisporus*.

### Analysis of general physicochemical properties

**Fig 4:**
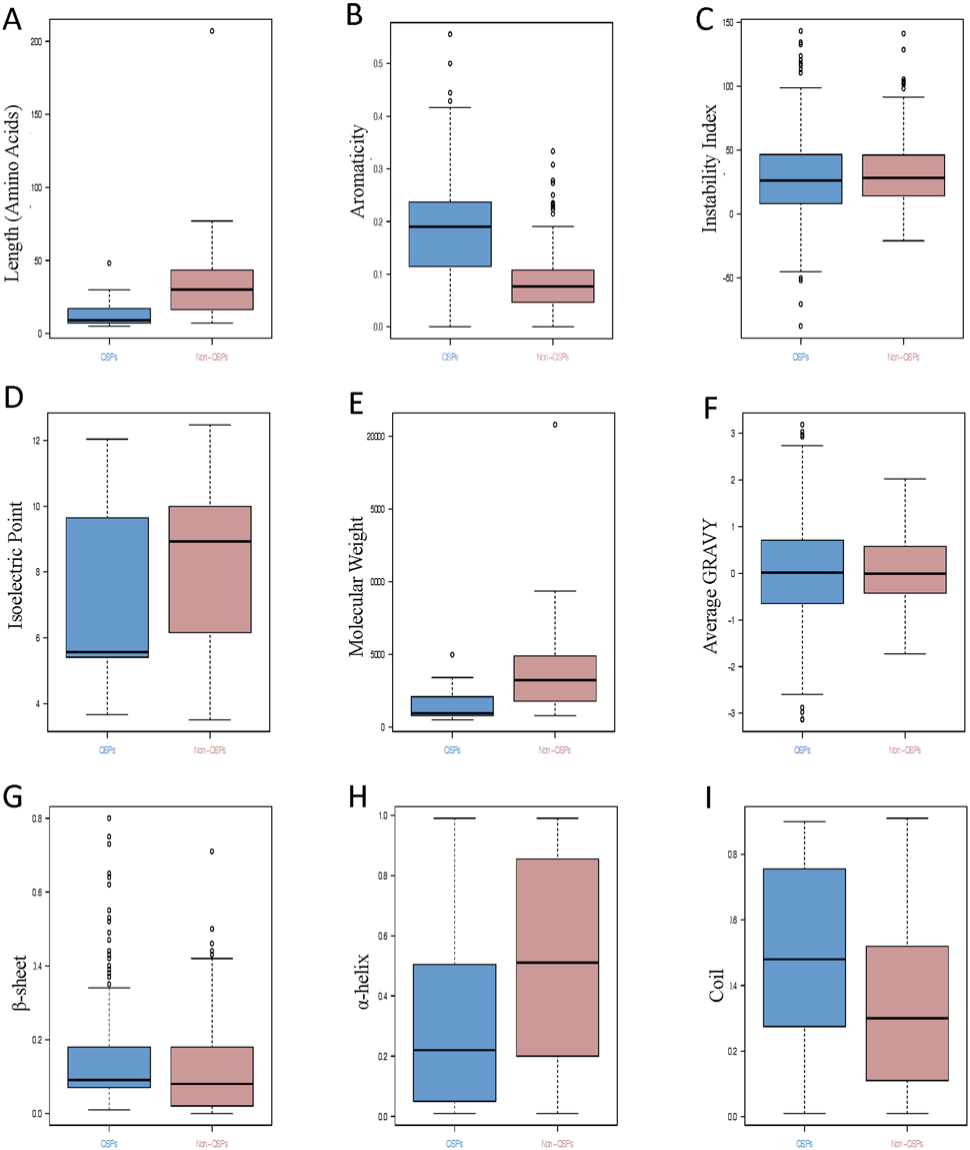
Statistical distribution of the physicochemical properties of Quorum sensing peptides (QSPs) and non-Quorum sensing peptides (non-QSPs). Each panel corresponds to respective parameter A) Length, B) Aromaticity, C) Instability Index, D) Isoelectric point, E) Molecular Weight, F) Grand Average of hydropathy (GRAVY), G) β- sheet, H) α-helix, and I) Random coil.

We have analyzed nine general physicochemical properties namely, length, aromaticity, instability index, isoelectric point, molecular weight, α-helix, β-sheet, coil and hydrophobicity. Statistical analysis of each parameter differentiating QSPs and non-QSPs was illustrated in Fig. 4. In case of length of QSPs, whiskers of boxplot represent that peptide ranged from 5 to 30 amino acids with an average of 11.5, while non-QSPs length varies from 7 to 77 amino acids having an average of 31.8 (Fig. 4A). So, the QSPs have lesser peptide length than non-QSPs. Aromaticity box-plot analysis indicated that QSPs were having more abundant aromatic amino acids than that of non-QSPs. QSPs have average aromaticity of 0.18 while for non-QSPs, the average aromaticity is 0.08 (Fig. 4B). A peptide is considered stable if its average instability index is less than 40[33]. Average instability index of QS and non-QSPs were 28.2 and 32.6, respectively. It portrayed that QSPs are comparatively stable than non-QSPs (Fig. 4C). Average isoelectric point of QS and non-QSPs were 7.1 and 8.3, respectively. It indicates that these peptides may bear net positive or negative charge if their pH is below or above their respective average isoelectric points (Fig. 4D). Average molecular weight of QSPs was 1322.9 while that of non-QSPs was 3489.7 (Fig. 4E). Maximum QSPs had a tendency to behave as hydrophobic in comparison to non- QSPs (Fig. 4F) as their GRAVY score was positive than non-QSPs. Each residue of QSPs and non-QSPs was categorized into α-helix, β-sheet or coil by predicting secondary structure. Box-plots of α-helix, β-sheet and coil between QSPs and non-QSPs (Figs. 4G, 4H and 4I) showed variance and thus indicate the importance of secondary structure in QSPs.

### Analysis of top performing physicochemical properties from SVM

Additionally, we have also analyzed best performing physicochemical indices (from AAindex) which mainly include secondary structure components like Normalized frequency of alpha-helix (NAGK730101), Average relative probability of helix (KANM800101), Normalized positional residue frequency at helix termini C" (AURR980118), Normalized positional residue frequency at helix termini N" (AURR980103), Normalized positional residue frequency at helix termini N4' (AURR980101), The Chou-Fasman parameter of the coil conformation (CHAM830101), Normalized frequency of N-terminal non beta region (CHOP780210) and Normalized frequency of beta-turn (CHOP780101). This further emphasizes and endorses conclusion from general physicochemical properties regarding importance of secondary structure in QSPs. Other features like pK-C (FASG760105), Radius of gyration of side chain (LEVM760105), Relative preference value at N2 (RICJ880105) and Polar requirement (WOEC730101) also displayed variance between QSPs and non-QSPs. Box-plots of these physicochemical indices are shown in Fig. B in S1 File.

### QSPpred algorithm development

#### Using negative dataset from UniProt

*Prediction of QSPs using 10-fold cross-validation technique*: 10-fold cross-validation was performed on QSP training/testing dataset (T^200p+200n^) using SVM by employing various peptide properties like amino acid compositions, binary patterns, physicochemical properties and their hybrids.

Composition (AAC+DPC) hybrid achieved an accuracy of 89.80% with MCC of 0.80. Positional (N5C5Bin) hybrid performed with an accuracy of 87.25% with MCC of 0.75. We checked the performance of each property from a total of 544 physicochemical properties available in the AAindex database [31]. An SVM model based on combined top 10 physicochemical properties (termed Physico) attained an accuracy of 93.00% with MCC 0.86. Further, we have also exploited hybrid approaches like AAC+DPC+N5C5Bin and AAC+DPC+N5C5Bin+Physico by combining different properties. These hybrids managed accuracy of 91.00% and 91.25% with MCC of 0.82 and 0.83, respectively. All the results are summarized in Table 3.

**Table 3:** Performance of SVM by employing distinct peptide properties during 10-fold cross validation using negative dataset from UniProt.

	Training/Testing dataset (T^200p+200n^)			Validation dataset (^V20p+20n^)		
Properties	Accuracy	MCC	ROC	Accuracy	MCC	ROC
AAC	89.00	0.78	0.95	82.50	0.65	0.94
DPC	91.00	0.82	0.95	87.50	0.75	0.94
AAC+DPC	89.80	0.80	0.96	85.71	0.72	0.95
N5Bin	84.25	0.69	0.92	85.00	0.70	0.95
C5Bin	86.00	0.72	0.92	77.50	0.55	0.92
N5C5Bin	87.25	0.75	0.95	90.00	0.80	0.93
Physico	93.00	0.86	0.98	90.00	0.82	0.97
AAC+DPC+N5C5Bin	91.00	0.82	0.96	92.50	0.86	0.95
AAC+DPC+N5C5Bin+Physico	**91.25**	**0.83**	**0.96**	**90.00**	**0.80**	**0.95**

AAC, Amino Acid Composition; DPC, Di Peptide Composition; N5AAC, Amino Acid Composition of 5 N-terminal residues; C5AAC, Amino Acid Composition of 5 C-terminal residues; N5Bin, Binary pattern of 5 N-terminal residues; C5Bin, Binary pattern of 5 C-terminal residues; N5C5Bin, Binary pattern of 5 N and 5 C terminal residues; Physico, top 10 physicochemical properties; SVM, Support Vector Machine; MCC, Mathew’s correlation coefficient; AUC, Area Under the curve;

*Performance evaluation on independent dataset*: As 10-fold cross-validation evaluation is not considered sufficient; therefore, an independent validation dataset V^20p+20n^ (not included anywhere in the SVM training) was used to assess models performance. Composition (AAC+DPC) model’s reached an accuracy of 88.10% and MCC of 0.78 whereas binary profile (N5C5Bin) got an accuracy of 87.50% and MCC of 0.75. Similarly, Physico model achieved an accuracy of 87.50% and MCC of 0.76. However, both hybrid models AAC+DPC+N5C5Bin and AAC+DPC+N5C5Bin+Physico performed slightly well with an accuracy of 90.00% with MCC of 0.82 as detailed in Table 3.

**Fig 5:**
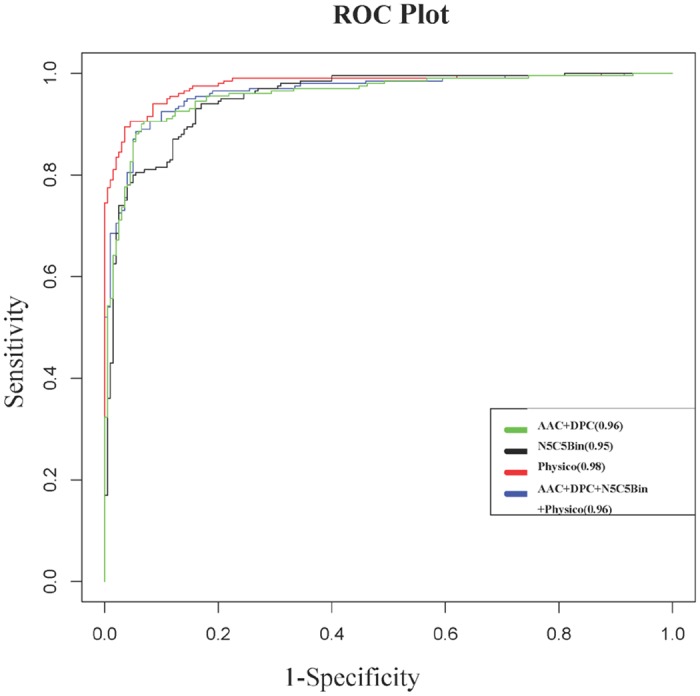
ROC curves of hybrid models AAC+DPC, N5C5Bin, Physico and AAC+DPC+Physico+N5C5Bin developed by SVM.

*ROC plot for validating threshold independent performance of hybrid models*: To check the threshold independent performance of various hybrid SVM models, ROC was plotted for AAC+DPC, N5C5Bin, Physico and AAC+DPC+Physico+N5C5Bin. ROC shows Area under the curve (AUC) between sensitivity and 1-specificity. These hybrid models contributed AUC of 0.96, 0.95, 0.98 and 0.96, respectively, as displayed in Fig. 5.

#### Using negative dataset from scrambled QSP sequences

*Performance evaluation of QSPs using negative dataset as scrambled sequences*: 10-fold cross validation was performed on QSPs and negative dataset by scrambling QSPs training/testing data set (T^200p+200sn^) on various peptide features like amino acid composition, dipeptide composition, binary patterns, physicochemical and their hybrids.

Composition peptide features, AAC and DPC achieved an accuracy of 50.50%, 77.50% and MCC of 0.03, 0.59 respectively. Binary pattern (N5C5Bin) attains accuracy of 80.75% and MCC of 0.63 while Physico gave accuracy of 100.00% with MCC of 1.00. Hybrid of all properties (AAC+DPC+N5C5Bin+Physico) reached an accuracy of 80.75% with MCC of 0.63 as detailed in Table 4.

**Table 4:** Performance of SVM by employing distinct peptide properties during 10-fold cross validation using negative dataset from scrambled QSP sequences

	Training/Testing dataset (T^200p+200sn^)			Validation dataset (^V20p+20sn^)		
Properties	Accuracy	MCC	ROC	Accuracy	MCC	ROC
AAC	50.50	0.03	0.51	50.00	0.00	0.50
DPC	77.50	0.59	0.84	75.00	0.58	0.80
N5C5Bin	**80.75**	**0.63**	**0.86**	**80.00**	**0.63**	**0.82**
Physico	100.00	1.00	0.50	50.00	0.00	0.50
AAC+DPC+N5C5Bin+Physico	80.75	0.63	0.86	50.00	0.00	0.50

AAC, Amino Acid Composition; DPC, Dipeptides Composition; N5C5Bin, 5N- and 5C-terminal binary pattern; Physico, hybrid of top 10 physicochemical properties; MCC, Mathew’s correlation coefficient; ROC, Reciever Operating Characterstic;

Independent dataset V^20p+20sn^ used to check the performance of models developed during training/testing. AAC, DPC, N5C5Bin, Physico and AAC+DPC+N5C5Bin+Physico achieved accuracies of 50.00%, 75.00%, 80.00%, 50.00%, 50.00% with MCC of 0.00, 0.58, 0.63, 0.00, 0.00 respectively as shown in Table 4.

### Comparison of QSPs, AVPs, AMPs and CPPs peptide sequences using amino acid composition

**Fig 6:**
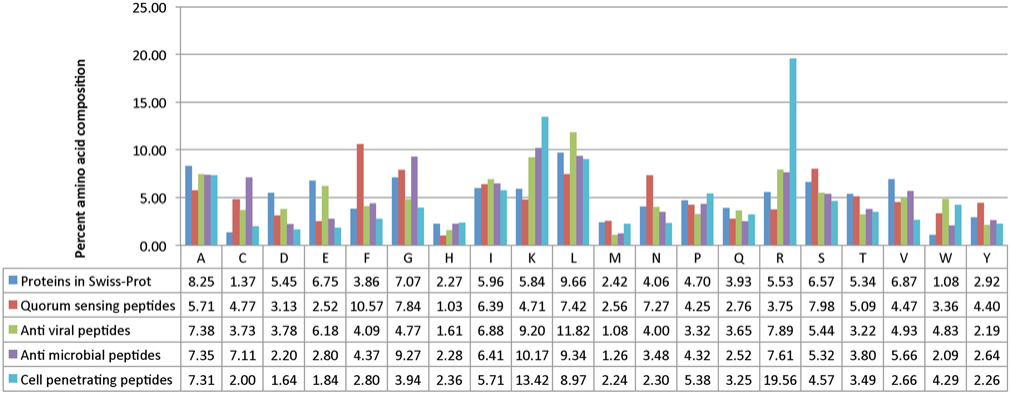
Amino acid compositional comparison of quorum sensing peptides (QSPs), antiviral peptides (AVPs), cell-penetrating peptides (CPPs) and antimicrobial peptides (AMPs) with overall amino acids composition in Swiss-Prot.

We compared different functional peptides *viz*. QSPs [7], AVPs [37], AMPs[25] and cell-penetrating peptides (CPPs) [38]. Percent amino acid compositions of above diverse peptides were compared with that of overall amino acid composition of proteins in Swiss-Prot as depicted in Fig. 6. We checked the differences in terms of fold change in all four categories of peptides as specified in Table A in S1 File.

Unique trends of amino acids occurrence in all four-category peptides were observed. Firstly, Cys and Trp were the most preferred residues in all. Largest fold changes were 5.19, 3.48, 2.72 and 1.46 for Cys residue in AMPs followed by QSPs, antiviral and CPPs, respectively as compared to Swiss-Prot. Whereas, Trp was abundant in AVPs followed by CPPs, QSPs and AMPs by 4.48, 3.97, 3.11 and 1.93 folds, respectively. Secondly, Asp and Glu were the most depleted residues among all four-category peptides. Asp residue was depleted in CPPs, AMPs, QSPs and AVPs by 3.33, 2.48, 1.74 and 1.44 folds, respectively. While, Glu composition decreased by 3.66, 2.68, 2.41 and 1.09 folds in CPPs, QSPs, AMPs and AVPs, respectively.

Interestingly, Lys and Arg were depleted by 1.24 and 1.48 fold, respectively, in QSPs. Whereas, both of these residues are highly abundant by 1.58 to 2.3 and 1.38 to 3.54 folds, respectively, among remaining three categories of peptides. Moreover, QSPs were enriched with Phe, Asn and Tyr residues by 2.74, 1.79 and 1.51 folds, respectively, in addition to Cys and Trp. It shows that all aromatic amino acids are favored in QSPs. However, in QSPs, apart from Asp and Glu, other residues like His, Val and Arg were depleted by 2.20, 1.54 and 1.48, respectively.

### Web server development

QSPpred web server hosts SVM based predictive models i.e. QSPepPred, QSPepDesign and QSPepMap utilizing compositional, binary, physicochemical features and their hybrids. *QSPepPred* predicts the extent of an input peptide (fasta or multifasta format) as QSP or non-QSP. *QSPepDesign* can design all possible single position mutants of a given peptide sequence and subsequently predict their QS status. *QSPepMap* helps to identify potential regions in protein, which may function as QSPs. In addition, it also includes various analysis tools like *QSMotifScan*, *MutGen*, *PhysicoProp* and *ProtFrag*.

## Discussion

QSPs play key role in numerous applications like infection by *Staphylococcal* species [39] and *E*. *faecalis* [14]; a potential role in oncology [40,41]; fermentation technology for the production of bioethanol by *Clostridium acetobutylicum* [42] and many more. Despite such significances of QSPs, there is no approach in the literature to identify QSPs. Therefore, we have performed analysis and prediction of QSPs as they are of immense importance in coordinating the behavior of Gram-positive bacteria.

Various peptide sequence properties like amino acid composition, position, motifs and physicochemical properties were analyzed for QSPs and non-QSPs. For the present study experimentally validated non-redundant positive data set were used.

Several important characteristics and properties were identified based on the QSPs analysis. Their compositional analysis concluded that they are rich in aromatic amino acids, namely, Phe, Trp and Tyr. We found small residues such as Asn, Pro and Ser at N-terminal of which Ser was also reported earlier at N-terminal in *S*.*mutans* JH1005 [43]. Conversely, larger amino acids like Trp, Phe, Lys, Gln were preferred at the C-terminal along with Cys, a small residue. Out of these, role of Trp and Cys were also highlighted in previous studies [44]. Furthermore, dipeptide composition analysis revealed Leu-Phe, Asn-Asn, Ile-Phe, Ser-Thr, Ser-Leu, Cys-Val, Pro-Cys, Val-Gly, and Phe-Phe as preferred consecutive residues.

Several identified QS motifs were searched in the PROSITE database. Interestingly, we discovered some of these motifs not only in the proteome of Gram-positive bacteria, but also in Gram-negative bacteria and archea. Both of these were reported to have quorum sensing through AHLs [45,46]. Presence of the predicted QSP motifs in Gram-negative bacteria and archea needs to be experimentally validated for their role in the quorum sensing.

Additionally, in the context to various physiochemical properties, like aromaticity, molecular weight and secondary structure etc., some of them majorly differentiate QSPs from non-QSPs. Moreover, the role of aromaticity is also affirmed from AAC analysis in which aromatic residues like Phe, Trp and Tyr are preferred in QSPs. Further, we observed that QSPs prefer secondary structure conformations (α-helix, coil and β-sheet). This observation was further endorsed by results of box-plot from top physicochemical indices extracted using SVM. However, the previous study [47] has shown the preference of α-helix by CSP in *S*. *pneumoniae*. On the other hand, CSP of *S*. *mutans* has been reported to be in random coil initially but adopted an α-helix conformation upon binding to the receptor [48,49].

On comparing QSPs, AVPs, AMPs and CPPs along with proteins in Swiss-Prot we observed that all categories of peptides have abundant Cys and Trp residues. These amino acids are important for peptides to be functional as Trp has been reported to have a role in binding sites [50,51] and anchoring [52]. While, Cys residue is reported to form disulfide bonds that increase activity [53] and stability [54] in secretory peptides.

On the basis of these analyzed QSPs features, we have developed “QSPpred”, a QSPs prediction algorithm using SVM. For this, two negative data sets (first from UniProt and second by scrambled QSP sequences) along with QSPs were employed. Our predictor achieved a high accuracy of 93.00% during 10-fold cross-validation and 90.00% on independent dataset using first approach. Performance of the first strategy was better than the second during 10-fold cross validation and also on independent validation data sets. The drawback in the performance of second strategy could be attributed to the fact that both QSPs and scrambled sequences as non-QSPs share same amino acid compositions. Hence, second strategy is unable to efficiently predict QSPs. We also explored the feasibility of BLAST similarity search to identify QSPs (accuracy of 77.50%). However, performance of our SVM based predictor is far better. Finally, QSPs analysis and their predictor “QSPpred” would accelerate research in the field of quorum sensing.

## Supporting Information

S1 File **Fig. A.** Amino acid compositional analysis of Quorum sensing peptides (QSPs). Comparison of percent amino acid composition of QSPs with proteins of Gram-negative bacteria, Gram-positive bacteria and total proteins in Swiss-Prot. **Fig. B**. Statistical distribution of physicochemical properties of Quorum sensing (QSPs) and non-Quorum sensing peptides (non-QSPs). **Table A.** Amino acid compositional comparison. Study of Quorum sensing peptides (QSPs), antiviral peptides (AVPs), antimicrobial peptides (AMPs) and Cell-penetrating peptides (CPPs) with reference to amino acid composition of complete Swiss-Prot proteins (on basis of fold change)
(DOC)

S2 File **Table B.** List of 220 Quorum sensing peptides. **Table C.** List of 220 non Quorum sensing peptides.
(XLS)
