# Supplementary material for: Prediction and Analysis of Quorum Sensing Peptides Based on Sequence Features
Source: PLoS One. 2015 Mar 17;10(3):e0120066. doi: 10.1371/journal.pone.0120066 (PMC4363368; doi:10.1371/journal.pone.0120066)
Supplement: S1 File — Fig. A. Amino acid compositional analysis of Quorum sensing peptides (QSPs). Comparison of percent amino acid composition of QSPs with proteins of Gram-negative bacteria, Gram-positive bacteria and total proteins in Swiss-Prot. Fig. B. Statistical distribution of physicochemical properties of Quorum sensing (QSPs) and non-Quorum sensing peptides (non-QSPs). Table A. Amino acid compositional comparison. Study of Quorum sensing peptides (QSPs), antiviral peptides (AVPs), antimicrobial peptides (AMPs) and Cell-penetrating peptides (CPPs) with reference to amino acid composition of complete Swiss-Prot proteins (on basis of fold change) (DOC) [file pone.0120066.s001.doc]

**Prediction and analysis of quorum sensing peptides based on sequence features**

Akanksha Rajput, Amit Kumar Gupta and Manoj Kumar*

**Supplementary information**

**Fig. A. Amino acid compositional analysis of Quorum sensing peptides (QSPs)**. Comparison of percent amino acid composition of QSPs with proteins of Gram-negative bacteria , Gram-positive bacteria and total proteins in Swiss-Prot.


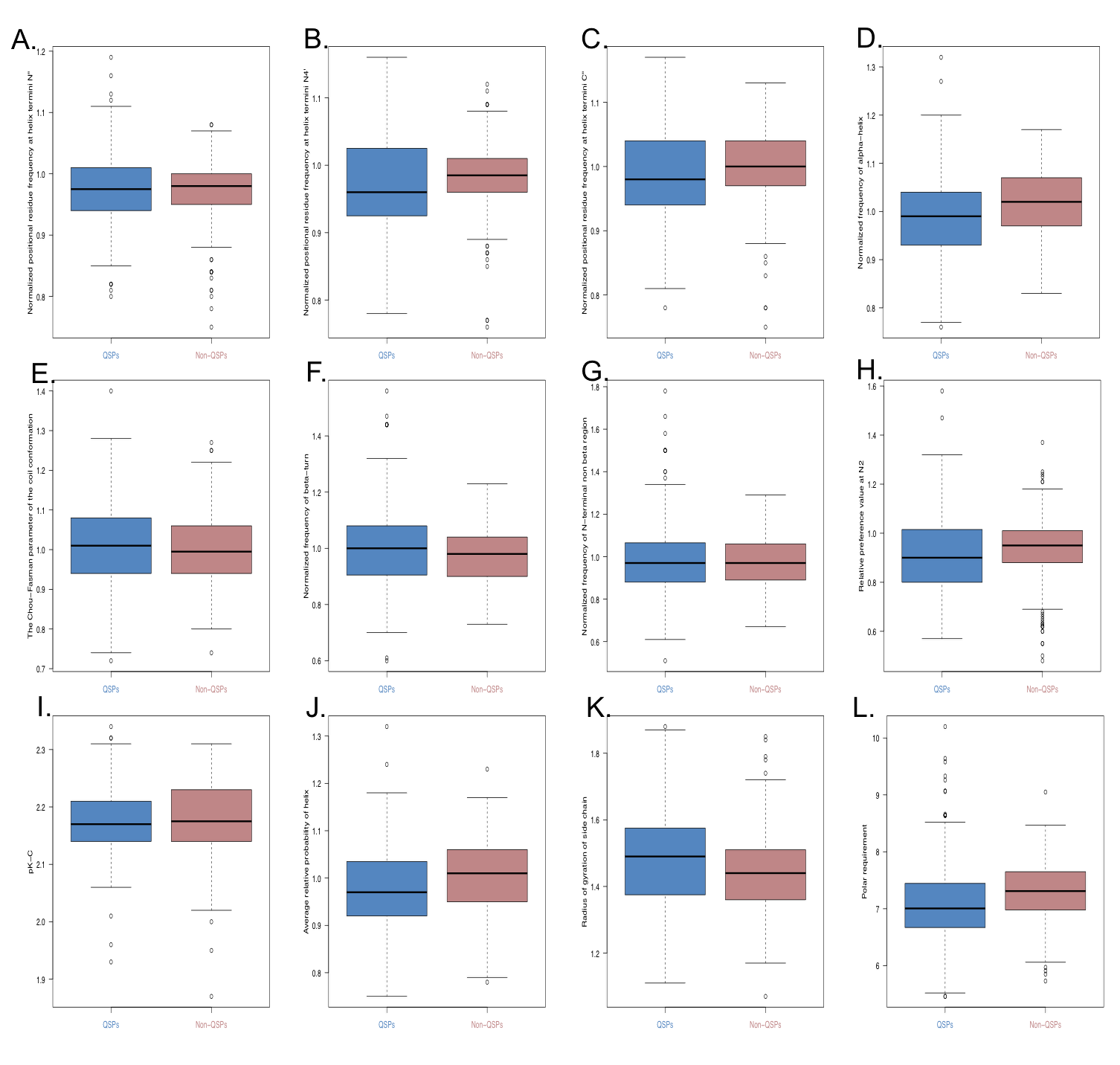


**Fig. B. Statistical distribution of physicochemical properties of Quorum sensing (QSPs) and non-Quorum sensing peptides (non-QSPs).** Box plot are shown of best physicochemical properties extracted from Support Vector Machine A) Normalized positional residue frequency at helix termini N" (AURR980103), B) Normalized positional residue frequency at helix termini N4' (AURR980101), C) Normalized positional residue frequency at helix termini C" (AURR980118), D) Normalized frequency of alpha-helix (NAGK730101), E) The Chou-Fasman parameter of the coil conformation (CHAM830101), F) Normalized frequency of beta-turn (CHOP780101), G) Normalized frequency of N-terminal non beta region (CHOP780210), H) Relative preference value at N2 (RICJ880105) I) pK-C (FASG760105), J) Average relative probability of helix (KANM800101), K) Radius of gyration of side chain (LEVM760105) and L) Polar requirement (WOEC730101).

**Table A. Amino acid compositional comparison.** Study of Quorum Sensing peptides (QSPs), antiviral peptides (AVPs), antimicrobial peptides (AMPs) and cell-penetrating peptides (CPPs) with reference to amino acid composition of complete Swiss-Prot proteins (on basis of fold change).

| **Amino Acid Residues** | **Quorum sensing peptides** | **Anti viral peptides** | **Anti microbial peptides** | **Cell penetrating peptides** |
| --- | --- | --- | --- | --- |
| A | 0.69 | 0.89 | 0.89 | 1.00 |
| **C** | **3.48** | **2.72** | **5.19** | **1.91** |
| **D** | **0.57** | **0.69** | **0.40** | **0.58** |
| **E** | **0.37** | **0.92** | **0.41** | **0.45** |
| F | 2.74 | 1.06 | 1.13 | 1.07 |
| G | 1.11 | 0.67 | 1.31 | 1.94 |
| H | 0.46 | 0.71 | 1.00 | 1.41 |
| I | 1.07 | 1.15 | 1.08 | 0.94 |
| K | 0.81 | 1.58 | 1.74 | 1.10 |
| L | 0.77 | 1.22 | 0.97 | 0.79 |
| M | 1.06 | 0.45 | 0.52 | 1.16 |
| N | 1.79 | 0.99 | 0.86 | 0.87 |
| P | 0.90 | 0.71 | 0.92 | 1.30 |
| Q | 0.70 | 0.93 | 0.64 | 0.69 |
| R | 0.68 | 1.43 | 1.38 | 0.97 |
| S | 1.21 | 0.83 | 0.81 | 0.98 |
| T | 0.95 | 0.60 | 0.71 | 1.18 |
| V | 0.65 | 0.72 | 0.82 | 1.14 |
| **W** | **3.11** | **4.48** | **1.93** | **0.43** |
| Y | 1.51 | 0.75 | 0.90 | 1.20 |
